# Oral Immunization of Rabbits with *S. enterica Typhimurium* Expressing *Neisseria gonorrhoeae* Filamentous Phage Φ6 Induces Bactericidal Antibodies Against *N. gonorrhoeae*

**DOI:** 10.1038/srep22549

**Published:** 2016-03-04

**Authors:** Andrzej Piekarowicz, Aneta Kłyż, Michał Majchrzak, Daniel C. Stein

**Affiliations:** 1 Department of Virology, Institute of Microbiology, Faculty of Biology, University of Warsaw, Warsaw, Poland; 2 University of Maryland, Department of Cell Biology and Molecular Genetics, College Park, MD 20742, USA.

## Abstract

All *Neisseria gonorrhoeae* strains whose DNA sequences have been determined possess filamentous phage DNA sequences. To ascertain if phage encoded proteins could form the basis of a gonococcal vaccine, rabbits were orally infected with *S. enterica* *Typhimurium* strain χ3987 harboring phagemid NgoΦ6 fm. The elicited sera contained large quantities of anti-phage IgG and IgA antibodies that bound to the surface of *N. gonorrhoeae* cells, as shown by indirect fluorescent analysis and flow cytometry. The elicited sera was able to bind to several phage proteins. The sera also had bactericidal activity. These data demonstrate that *N. gonorrhoeae* filamentous phage can induce antibodies with anti-gonococcal activity and that phage proteins may be a candidate for vaccine development.

*Neisseria gonorrhoeae* (GC), responsible for the sexually transmitted infection gonorrhea, can infect the reproductive tract of women, and if the infection is left untreated, it can lead to pelvic inflammatory disease (PID) or infertility, and can increase the risk of ectopic pregnancy. GC infections also increase the risk of acquiring HIV^1^. While these factors represent important health issues and should justify the development of a vaccine, the recent emergence of ceftriaxone-resistant *N. gonorrhoeae*^2^ raises new concerns for controlling gonorrhea because ceftriaxone is widely recommended as the first-line treatment for gonorrhea around the world. In 2008, calculations of the cost of GC infections in women, which are likely to underestimate the true cost of diagnosing and treating infertility and its sequelae, exceeded $5 billion per year^3^. A vaccine that could reduce carriage or prevent reproductive damage in women would be an important addition to health care for women.

Tapchaisri and Sirisinha^4^ showed that most male patients with acute gonorrhea develop anti-GC antibody within 2 wks of infection. However, antibody levels declined to background in 1 to 2 months. This lack of immunological memory suggests a carbohydrate antigen (i.e. lipooligosaccharide) as the primary immunogen. Kasper *et al.*^5^ found a bactericidal antibody response in about one-third of uncomplicated infections, but in only 5% of women with salpingitis, suggesting that bactericidal antibodies protect against salpingitis. Furthermore, convalescent sera showed a fourfold rise in bactericidal titers in women with severe salpingitis. Hence, clinical data suggests that a GC vaccine could significantly impact human health. While there is no vaccine against *N. gonorrhoeae*, several gonococcal components have been tested in humans (pilin^6^), or mice (NspA^7^, PorB^8^,^9^, transferrin binding protein^10^, and an oligosaccharide mimetic^11^) to name a few.

Characterization of filamentous phages led to the development of phage display technology, where a range of peptides and proteins have been expressed as fusions to phage coat proteins and displayed on the viral surface^12^. Phage display was adapted for the construction of vaccines using recombinant filamentous phage as carriers of immunogenic peptides or peptide mimotope fusion peptides. Recombinant filamentous phage stimulate strong B and T cells responses even without adjuvants^13^,^14^,^15^,^16^,^17^,^18^. Phage particles displaying peptides or peptide mimotopes of pathogens or tumors induce specific antibodies^19^. A few published examples of phage-based therapeutics include preparations for the treatment of herpes simplex virus^20^, HIV^21^, Alzheimer’s disease^22^, melanoma^23^, and HBV^24^. Furthermore, phage can be designed to specifically target antigen-presenting cells inducing potent cellular immunity^25^. Taken as a whole, the particulate nature, size, shape and composition of filamentous phage are characteristics of a vaccine carrier with strong and long-lasting immunogenic potential. Importantly, filamentous phage have been used experimentally in humans with the approval of the FDA with no apparent side effects^26^, indicating their safety.

All *N. gonorrhoeae* genomes characterized to date encode several filamentous phage whose DNA and protein sequences show ~95% identity^27^,^28^. Because the filamentous phage replication cycle is conserved among most of these phage in other species^29^, this suggests that the assembly and structural proteins should be present on the surface of GC. This makes them potential targets for specific antibodies. These facts suggest that filamentous phage proteins could be the basis of a gonococcal vaccine. Our discovery that *N. gonorrhoeae* filamentous phage can replicate and be stably maintained in different Gram-negative bacteria^27^ suggested that this could allow for a novel way of delivering phage particles, using live non-pathogenic bacteria as the delivery vehicle.

Live bacterial vaccine vectors such as attenuated human intestinal bacteria like *Salmonella*, *Shigella* or *Listeria* have been studied for mucosal immunization for the prevention of different infectious diseases^30^,^31^,^32^. These microorganisms, when delivered through the oral route, can cross the lumen of the gut and be taken up by macrophages and dendritic cells at local sites, which results in the stimulation of humoral as well as cell-mediated and mucosal immune responses. Here we evaluate the effectiveness of phage NgoΦ6 as a potential immunogen delivered by the *S. enterica* χ3987 Typhimurium strain to induce anti-gonococcal antibodies. To our knowledge, this is the first application of using “wild type” filamentous phage where native phage proteins serve as the immunizing antigen.

## Results

To form an NgoΦ6-based vaccine*, S. enterica* χ3987 ser. Typhimurium was transformed with pBS::Φ6 and the resulting ampicillin resistant colonies tested for the presence of pBS::Φ6 and production of progeny phagemid particles. All colonies tested contained this phagemid and were able to produce phage (data not shown). One of these colonies, designated as STΦ6, was used in further experiments.

The properties of the 11 annotated open reading frames found in NgoΦ6 in the FA1090 genome (GeneBank accession number AE004969.1) are described in Table 1. The gene *orf9* (NGO1138) is located on a region of the phage genome where genes encoding proteins responsible for assembly and release of phage from the cell are located. ORF9 belongs to the pfam 5707 family of proteins and is structurally and functionally homologous to the Zot protein of CTXΦ (16% identity and 39% positives over 209 residues^33^) and like Zot, is required for the release of progeny phage particles (data not shown).

**Figure 1:**
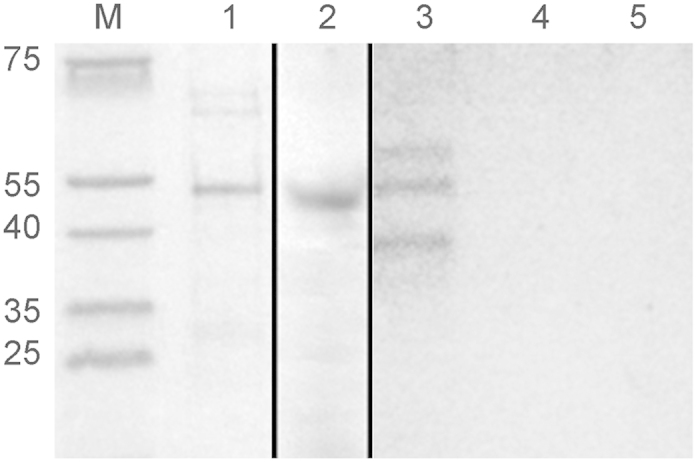
Localization of ORF9 in cellular compartments. An aliquot of His-tagged ORF9 protein purified by metal affinity chromatography was separated on a 15% SDS-PAGE gel and stained with Coomassie brilliant blue R250. Lane M, PageRuler prestained protein ladder; lane 1, ORF9 protein. To demonstrate the mobility of ORF9, western blot analysis (lane 2) was performed using a mouse monoclonal anti-His IgG-alkaline phosphatase conjugate antibody to detect the His-tag found on ORF9. For analysis of the subcellular localization of ORF9 protein in *E. coli* Top10 cells with Ngoϕ6fm, cells were fractionated, enriched by chromatography on a metal affinity column and aliquots from the outer membrane (lane 3), cytoplasm-periplasm (lane 4) and inner membrane (lane 5) separated on an SDS-PAGE gel and stained with Coomassie brilliant blue R250. For this figure, lanes M and 1 were analyzed on one gel, lane 2 on a second gel, and lane 3–5 on a third gel. Gels 2 and 3 were run simultaneously, with one gel used for Coomassie staining and the second gel used for transfer to nitrocellulose.

Judging by the genomic similarity of NgoΦ6 to other filamentous phages, the ORF9 protein (predicted molecular weight of 40.8 kDa) should be present in the outer membrane of bacterial cells during assembly and release of progeny phage and should be a target for anti-phage antibodies. We determined the cellular localization of ORF9 in *E. coli* by performing cell fractionation, followed by SDS-PAGE analysis of the samples. We observed a protein band that is consistent with the size of ORF9. Localization of ORF9 in outer membrane preparations of *E. coli* (pBS::Φ6) (Fig. 1) cells suggests that this protein will have the same localization in *N. gonorrhoeae* cells. Because the 13 gonococcal strains whose genomes have been sequenced at the Broad Institute (https://www.broadinstitute.org/) contain filamentous phage sequences with significant homology to NgoΦ6, and the ORF9 sequences are ~99% identical across all 13 isolates at the DNA level (DCS unpublished data), this suggests that anti-ORF9 antibodies should react with all gonococcal strains and could form the basis of a gonococcal vaccine.

### *Salmonella enterica* sv. Typhimurium χ3987 carrying pBS::Φ6 (STΦ6) elicits antibodies reactive with NgoΦ6 ORF9

**Figure 2:**
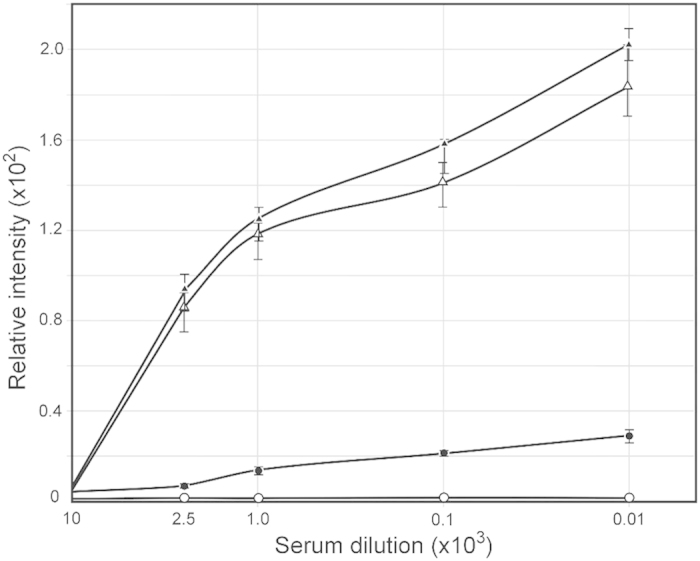
Serum IgG antibodies levels specific for *N. gonorrhoeae* ORF9 protein. Rabbits were immunized *via* oral administration of *S. enterica* sv. Typhimurium χ3987 carrying pBLΦ6. Sera obtained at day 38, 66 and 87 was analyzed by quantitative dot ELISA. His-tagged ORF9 protein (5 μg/ml) was affixed to nitrocellulose filters and incubated with different dilutions of rabbit sera. Binding of anti ORF9 IgG was detected with goat anti-rabbit IgG-alkaline phosphatase conjugate. Data are presented as mean ± S.D. of two separate experiments each performed in duplicate. The intensity of the color of the each spot was expressed as the change of the spot intensity compare to the negative control where spotting of ORF9 was omitted. For each point 4 spots were analyzed. The lines represent: o – o, pre-immunized sera; ●- ●, day 38 sera; Δ- Δ, day 66 sera; and ▲-▲, day 87 sera.

To determine if STΦ6 can induce the production of antibodies recognizing ORF9, approximately 10^8^ CFU of STΦ6 was used to immunize rabbits. The amount of IgG specific for ORF9 was determined by quantitative spot ELISA and the data showed in a dose dependent manner a high level of reactivity to purified ORF9 with sera from immunized rabbits. The level of antibodies against ORF9 increased after the first immunization and showed a booster effect with additional immunizations (Fig. 2). For negative controls, we omitted on the membrane either ORF9 protein or bacterial cells. In a separate control experiment, pre-immunized rabbit sera was used and the values obtained were subtracted from the values obtained with immunized sera. Ab levels against ORF9 in serum from rabbits immunized with *S. typhimurium* lacking pBS::Φ6 were the same as in pre immune serum.

**Figure 3:**
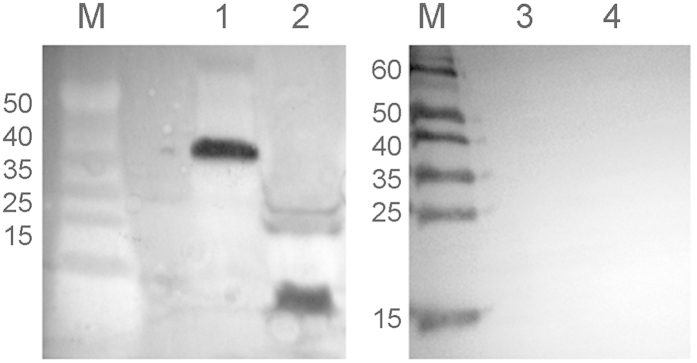
Reactivity of rabbit’s sera obtained after immunization with *S. enterica* sv. Typhimurium χ3987 (NgoΦ6fm) and *N. gonorrhoeae* proteins. Phage particles were separated on SDS-PAGE gels and subjected to Western blot analysis. In lanes 1 and 3, extracts were derived from broth-grown gonococci. In lanes 2 and 4, extracts were from purified phage. Lanes 1 and 2 were incubated with day 66 sera and lanes 3 and 4 were incubated with pre-immunized rabbit sera.

We performed a western blot to determine which phage proteins elicited antibody. In a whole cell extract of FA1090, we expected only the phage export protein ORF9 was expected to be present in significant quantities. In our purified phage, we expected the structural proteins of the phage to be present, but ORF 9 to be absent. The data in Fig. 3, lane 1 indicate that the elicited antibody bound to a single protein with a molecular mass consistent in size with ORF9 in the whole cell extracts. The data in Fig. 3 lane 2 indicate that antibodies produced by immunized rabbits react with numerous phage proteins. Sera from pre-immunized rabbits did not react with phage proteins (Fig. 3, lane 3 and 4) in either extract. Among the proteins present in phage particles, reactivity with sera was observed with different intensities.

### Polyclonal antisera generated by STΦ6 produce IgG and IgA antibodies against *Neisseria gonorrhoeae*

**Figure 4:**
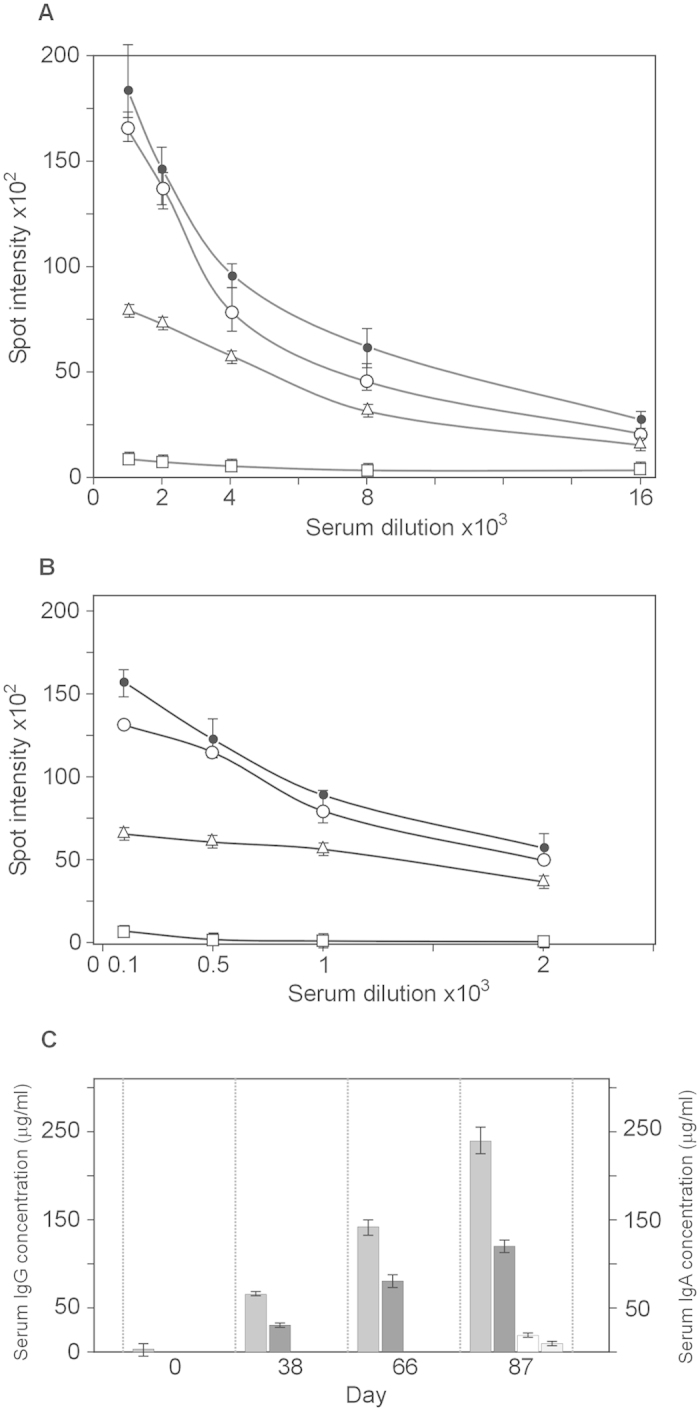
Serum IgG and IgA antibodies levels. Sera were analyzed by quantitative dot ELISA. *N. gonorrhoeae* cells (2 μl of 10^8^ cells/ml) diluted in PBS were spotted onto a nitrocellulose strip and allowed to dry. Panel (**A**) Titers of IgG polyclonal antibodies collected on days 0, 38, 66 and 87 after immunization. The intensity of the color of the each spot was expressed as the change of the spot intensity compare to the negative control where spotting of bacteria was omitted. For each point 4 spots were analyzed. Line: ●-●, day 87, o- o, day 66, Δ- Δ day 38, □- □, day 0). Panel (**B**) Titers of IgA polyclonal. The intensity of the color of the each spot was expressed as the change of the spot intensity compare to the negative control where spotting of bacteria was omitted. For each point 4 spots were analyzed. Line: ●-●, day 87, o- o, day 66, Δ- Δ day 38, □- □, day 0). Panel (**C**) The amount of antibodies was determined by comparing the optical density of specific anti-*N. gonorrhoeae* antibodies bound to the spots as presented in Panel **A**,**B** to a standard curve obtained with known quantities of purified mouse IgG reference antibodies. Standard curves were prepared by quantitative spot ELISA method. The amount of protein was quantified using GeneTools GBox (Syngen) program and expressed as the intensity of spot versus concentration of IgG or IgA protein. Since the measurement range of the ELISA dot was between dilution 2000 and 4000 the final determination of IgG concentration in all sera tested was based on of spot intensity values in this range. Determination of the IgG and IgA level against *N. gonorrhoeae* FA1090ΔФ6789 strain was based on similar experiments as in Panel A and Panel B (data not presented). Grey rectangle; anti-*N. gonorrhoeae* IgG, dark grey rectangle; anti-*N. gonorrhoeae* IgA, open rectangle; anti FA1090ΔФ6789 IgG, light rectangle; anti- FA1090ΔФ6789 IgA.

We determined the isotype of antibodies generated in rabbits immunized with STΦ6. The data presented in Fig. 4 show that oral immunization induced both IgG and IgA specific antibodies. Generation of both types of antibodies also showed a booster effect (Fig. 4A,B). The observed titer of IgG antibodies (about 250 μg/ml) (Fig. 4C) was higher than IgA antibodies (about 120 μg/ml) (Fig. 4C). The level of IgG and IgA antibodies was similar to the level of anti ORF9 antibodies (data not shown). The binding of antibodies to gonococcal cells was strongly dependent on the ability of these cells to produce filamentous phages since the mutant strain FA1090ΔФ6789 bound IgG antibodies at the level not exceeding 10% of binding by wild type strain (Fig. 4C). The level of antibodies binding to the whole cells of *N. gonorrhoeae* in pre-immunized rabbits was <5% of the maximal level of antibodies in immunized rabbit sera.

### Antibodies bind to whole gonococci

**Figure 5:**
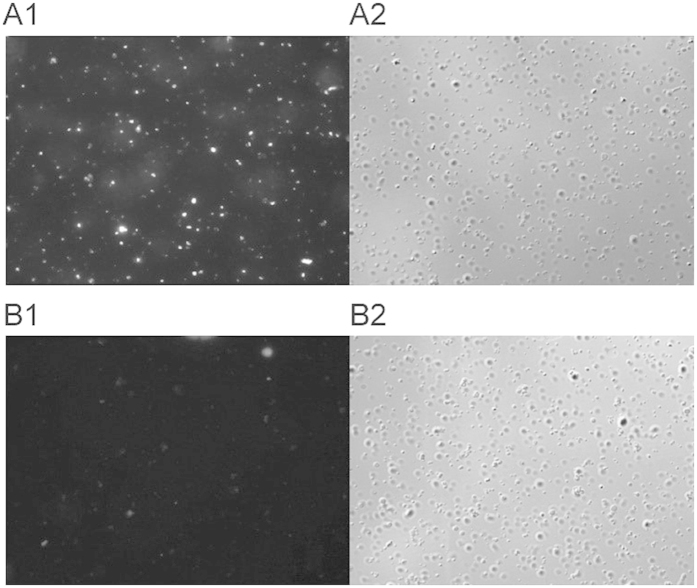
Immunofluorescent staining of *N. gonorrhoeae* FA1090. Bacterial cells (2 × 10^8^) were probed with rabbit sera immunized with *S. enterica* sv. Typhimurium χ3987 (NgoΦ6fm) obtained with day 66 sera (Panel **A1**) or prebleed sera (panel **B1**). Binding was detected by reaction with an AlexaFluor Cy3 goat anti-rabbit IgG (Invitrogen, Carlsbad, California) secondary antibody (Molecular Probes). Nomarski images (Panels **A2**,**B2**) correspond to the same fields as (**A1**,**B1**).

We determined that the elicited antibody was able to bind to intact gonococci by performing indirect immunofluorescence. The data in Fig. 5 illustrate that while the majority of GC appear to bind the antibody, the apparent fluorescence varied markedly among strains, probably reflecting the different degrees of ORF9 expression in individual bacteria.

**Figure 6:**
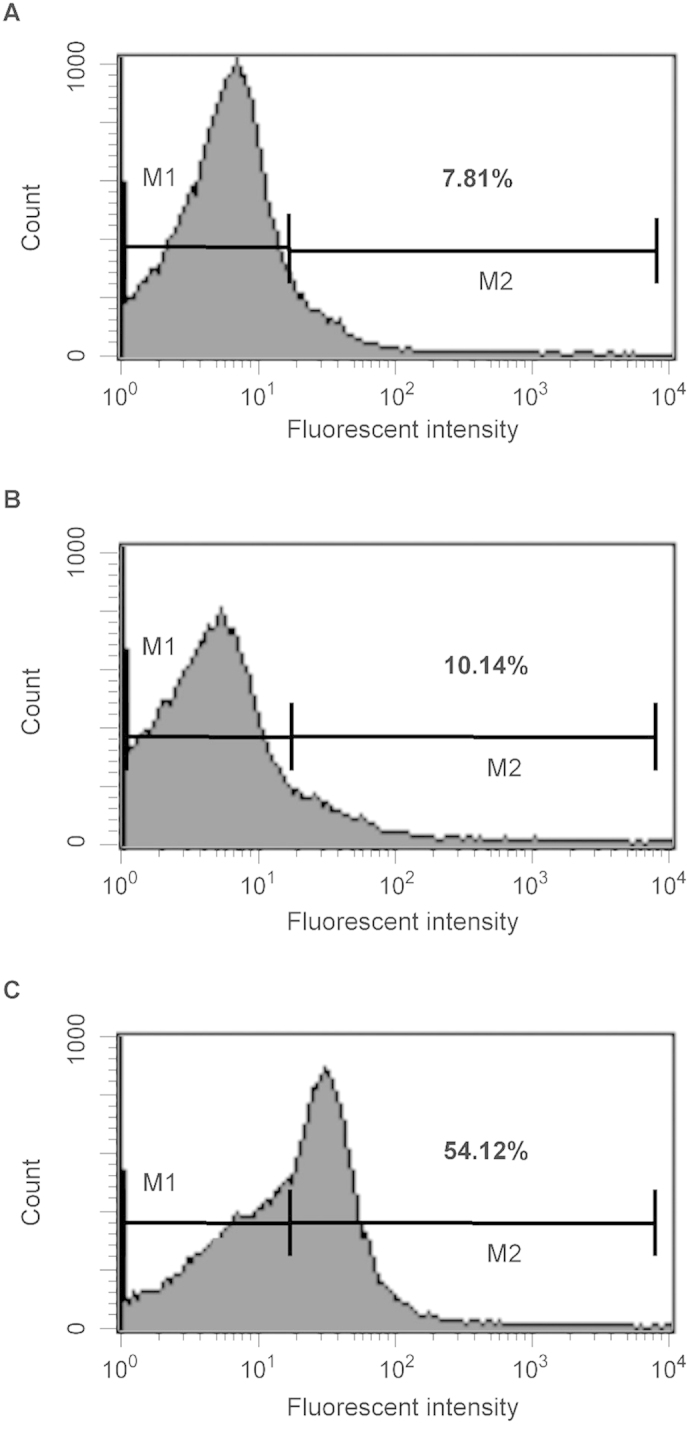
Flow cytometry analysis of antibody binding to *N. gonorrhoeae.* FA1090 cells were treated with buffer (panel **A**) pre-immunization sera (dilution 1:500) (panel **B**) or immunized sera (dilution 1:500) obtained after 66 day (panel **C**) followed by treatment with Cy3 goat anti-rabbit IgG (Life Technologies). The bacteria were analyzed by using FACS Calibur flow cytometer. Data were analyzed with CellQuest. Shown are representative histograms from three independent experiments. The bar in the figure represents the gate used to measure binding efficiency. The number in each figure corresponds to the percentage of the population that bound antibody.

In an effort to quantify the percentage of cells binding antibody, we measured the binding by flow cytometry. The data in Fig. 6 show a marked shift in the binding profile. Using the gating shown in the figure, 52% of cell population bound significant levels of IgG present in immunized sera (Fig. 6C); minimal binding was observed with preimmunized sera (Fig. 6B). A similar level of background binding was observed in a strain lacking filamentous phage genomes (data not shown). The results obtained with fixed cells or live cells generated the same Flow profiles, indicating that fixation did not mask or alter epitopes to which antibodies could bind (data not shown).

### Serum bactericidal activity

**Figure 7:**
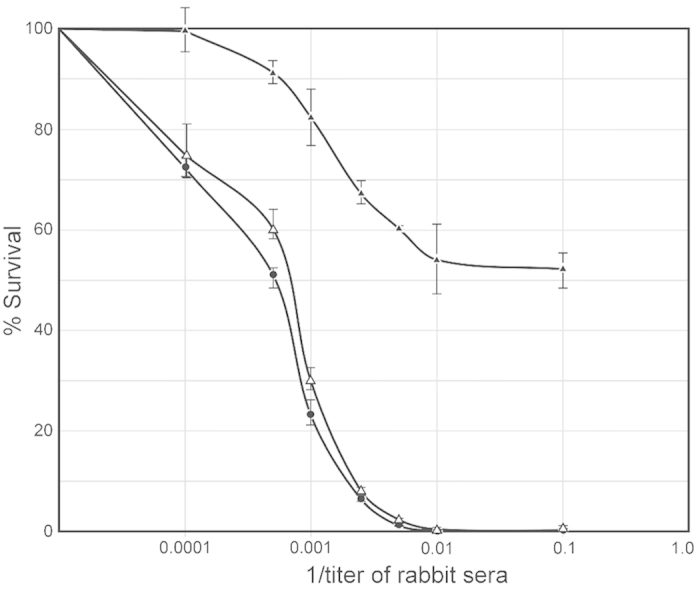
Bactericidal properties of elicited antibody. *N. gonorrhoeae*-specific bactericidal activity of sera from immunized rabbits was detected using an antibody complement-mediated bactericidal assay. Serum samples (heat inactivated at 56 °C for 30 min.) were mixed with gonococcal cells (3 × 10^4^ CFU/ml) and incubated at 37 °C and for 15 min. Normal undiluted human serum (final concentration of 4%) was added to the mixtures as a complement source and incubated for an additional 45 mins. The number colony forming units from these mixtures containing the immunized serum were counted and compared to those from negative controls (gonococci incubated with normal rabbit serum). *N. gonorrhoeae* MS11; ● - ●, *N. gonorrhoeae* FA62: Δ – Δ, *N. gonorrhoeae* FA1090: ▲ - ▲.

The results of our ELISA experiments with whole gonococcal cells, our ability to detect binding by as indirect fluorescent microscopy and our flow cytometry experiments showed that serum IgG bound the native bacterial cells. To test whether STΦ6 elicits bactericidal antibodies, we performed a bactericidal assay using pooled human sera as a complement source. We used day 66 sera from the rabbits and determined relative bactericidal activities against homologous and heterologous gonococcal strains. We determined that the addition of 8% complement did not result in detectable killing of the three test strains (data not shown). The data in Fig. 7 indicate that the elicited antibody was able to kill the three strains to differing degrees.

## Discussion

Numerous gonococcal surface proteins have been tested in various models for vaccine potential, including PilC, PilQ, Opa, AniA, TdfJ, PorB, Lst, Tbp,TbpA, 2C epitope, OmpA and OpcA (see review by Jerse *et al.*^34^). However, none of these studies considered the vaccine potential of antigens derived from the filamentous phages, even though these proteins are highly conserved and encoded by all examined *N. gonorrhoeae* strains. Since the invention of phage display technology^35^, filamentous phage have been adapted for use in many biological processes, including screening for ligands^36^ developing new drugs^37^, diagnosing diseases^38^ or designing vaccines^39^. When used for designing vaccines, the filamentous phage have served as a delivery vector for immunogenic peptides as they provide benefits such as high immunogenicity, low production costs and, high stability^19^.

During filamentous phage assembly, various forms of phage proteins need to be transferred to the host membrane to complete the assembly of progeny phages^40^. For M13, the phage proteins pIII and pVIII use their own signal peptides for their delivery into the cell membrane while for other fusion peptide it is necessary to use other signal peptides, mainly from outer membrane proteins^41^. The discovery that all *N. gonorrhoeae* strains are lysogens for several closely related filamentous phages suggested that they might serve as a unique source of conserved antigens for potential vaccine development. Since filamentous phage show strong stimulation of T helper cells^13^ without adjuvants^17^,^18^ and these phage are ubiquitous in *N. gonorrhoeae* where the phage proteins show little antigenic variation, it suggests that they may form a new paradigm for gonococcal vaccine development.

The data presented in this paper show that rabbits immunized with *Salmonella enterica* var. Tyhimurium χ3987 expressing NgoΦ6 generated very high levels of antibodies recognizing gonococcal cells. We demonstrated this binding by spot ELISA, fluorescent microscopy and flow cytometry. In other phage, orthologs of this protein play a critical role in the release of phage from the infected bacterial cells^29^. This protein has significant homology with zonula occludens toxin (ZOT) (data not shown), a 399 amino acid multifunctional enterotoxin encoded by phage CTXΦ of *Vibrio cholerae* that increases the permeability of small intestinal mucosal membrane by opening intercellular junctions^42^. Moreover, the Zot protein encoded by CTXΦ can act as a powerful mucosal adjuvant and induce specific IgG and IgA antibodies in vaginal and intestinal secretions^43^,^44^. Our unpublished results suggest that ORF9 plays a similar important role in the pathogenicity of gonococci, altering the organization of the tight junction. Similar to the Zot protein, ORF9 protein is toxic for a variety of human tissue culture cells (data not shown). While the use of a toxic protein in a vaccine may seem contraindicated, toxicity was observed only at very high protein concentrations. Blocking ORF9 activity would have very important consequences for gonococcal infections by potentially preventing tissue damage. This assumption is substantiated by the observation that sera from women infected with gonococci show the presence of antibodies against ORF9 (unpublished observations).

A second important property of sera obtained after immunization of rabbits with *S. enterica* var.Thyphimurium χ3987 carrying NgoΦ6fm was that they elicited very high level of specific IgG and IgA antibodies and showed bactericidal activity. Immunization strategies to elicit antibodies in the secretions of the genital tract is a challenge to gonorrhea vaccine development^34^. Most efforts to develop a vaccine have focused on conventional parenteral immunization, which generates circulating, predominantly IgG antibodies, but is generally ineffective at inducing secretory (s) IgA at mucosal surfaces.

For our studies we choose an attenuated strain of *Salmonella enterica* var. Tyhimurium χ3987, as this strain has been used to deliver heterologous antigens^45^. In addition, this strain can be delivered orally, facilitating vaccine delivery. We have shown that this attenuated *Salmonella* strain carrying NgoΦ6fm administrated orally can be an efficient vector for these antigens to induce an immune response against *N. gonorrhoeae*. Altogether, our results demonstrated that NgoΦ6fm can serve as efficient antigen delivery system and has the potential to form a basis for a vaccine against *N. gonorrhoeae*

Development of a vaccine that can prevent gonorrhea faces many hurdles. With the lack of understanding of the immunological basis of acquired immunity in humans, it is unclear what correlates of protection can serve as a basis for vaccine licensure. It is possible that the vaccine that we have described will not be effective at preventing gonococcal colonization of the female reproductive track. However, would a vaccine that could prevent disease sequelae such as PID be acceptable? It is likely that a successful vaccine will require multiple components and approaches. Using native bacteriophage as an immunogen could allow for grafting important epitopes into the virion. Much still needs to be done.

## Materials and Methods

### Bacterial strains, plasmids, phages, and growth conditions

*Escherichia coli* strain DH5α, F^−^ φ80*lacZ*Δ*M*15Δ(*lacZYA*-*argF*)*U*169 *recA1 endA1 hsdR17*(r_K_− m_K_ + ) *phoA supE44* λ-*thi*-*1 gyrA96 relA1* and *E. coli* strain BL21(DE3) [F− *ompT gal dcm lon hsdS*_B_ (r_B_− m_B_−) λ(DE3)] (Novagen) were grown in Luria-Bertani broth (LB) at 37 °C or 30 °C. Antibiotics (kanamycin, ampicillin and spectinomycin) were used at the final concentration of 50 μg/ml and chloramphenicol at the final concentration of 10 μg/ml. *Salmonella enterica* sv. Typhimurium χ3987^46^ obtained from Roy Curtiss III was grown in Luria-Bertani broth (LB) in the presence of diaminopimelic acid (DAP) (100 μg/ml final concentration). *N. gonorrhoeae* strains FA1090 (obtained from Dr. W. Shafer at Emory University, Atlanta, GA), MS11 (obtained from Dr. H. Schneider at WRAIR, Washington, DC), F62 (obtained from Dr. P. F. Sparling, University of North Carolina, Chapel Hill, NC) and a mutant of FA1090 where the genomes of Ngoϕ6, Ngoϕ7, Ngoϕ8 and Ngoϕ9 were deleted (this study) were used for these studies. *Neisseria* strains were grown in phosphate-buffered gonococcal medium (Difco) supplemented with 20 mM glucose and growth supplements^47^ either in broth with the addition of 0.042% NaHCO_3_ or on agar at 37 °C in an incubator with 5% CO_2_. Plasmid pBluescript KS(+)^48^ was purchased from MBI Fermentas. Phagemid pBL::Φ6 construction and properties were described previously^27^, pHP45^49^, pKRP11^50^ and pACYC184 (Stratagene) were used as a source of spectinomycin, kanamycin and chloramphenicol cassettes respectively.

### Enzymes and chemicals

Restriction enzymes were purchased from MBI Fermentas and New England BioLabs. T4 DNA ligase, Pfu DNA, Tag polymerases, and DNA and protein size markers were purchased from MBI Fermentas. Kits for DNA purification and plasmid DNA isolation were purchased from A&A Biotechnology (Gdansk, Poland). All chemicals used were reagent grade or better and were obtained from Sigma-Aldrich (St. Louis, MO), unless otherwise noted.

### DNA manipulations

Genomic DNA was extracted from cultures using the Genomic Mini DNA purification kit (ABA Biotechnologies). Plasmid DNA was purified using the GeneJet Plasmid Miniprep kit (Fermentas), and PCR products were purified using the GeneJet PCR purification kit (Fermentas) according to the manufacturer’s instructions. Phage and phagemid DNAs were isolated using the QIprep Spin M13 kit (Qiagen). Testing for the presence of the prophage sequences in genomic or phage DNAs by PCR was performed with primers described in supplemental Table 1. PCRs were performed in 50-μl reaction mixtures containing 300 nM of the forward and reverse primers, 200 μM (each) deoxynucleoside triphosphates (Fermentas), 0.5 units of Taq or Pfu polymerases (Fermentas) in the supplier’s buffer, and 100 ng of DNA as the template. Reactions were performed in an MJ Mini thermocycler (Bio-Rad). The specificity of the PCR products was confirmed by DNA sequencing of the amplicons. All routine cloning procedures were carried out in accordance with protocols described by Sambrook and Russell^51^. PCR products and other DNA samples were subjected to electrophoresis though 1% agarose gels and stained with ethidium bromide.

### Transformation

Transformation-competent *E. coli* and *Salmonella* cells were prepared according to a procedure described previously by Inoue *et al.*^52^ and stored at −80 °C. To prepare cells for transformation, cells were thawed on ice, DNA added, and the mixture incubated on ice for 10 min. The bacteria were heat shocked at 37 °C for 2 min, the total volume in the tube was increased to 1 ml by the addition of LB broth, and the transformation mixture incubated at 37 °C for 30 min to 1 h to allow the bacteria to recover and begin expressing antibiotic resistance proteins. Transformed bacteria were plated onto LB agar plates containing appropriate antibiotics and, if necessary, X-Gal.

For the transformation of *N. gonorrhoeae*, piliated bacteria were resuspended to a light turbidity (1 × 10^7^ CFU/ml) in 1 ml of gonococcal medium base lacking agar (GCP) containing 10 mM MgCl_2_, Kellogg’s supplement^47^ (GCK), and 0.42% NaHCO_2._ DNA was added and the culture incubated at 37 °C with shaking for 2 to 6 h. Bacteria were plated onto GCK agar plates containing the appropriate antibiotic and incubated for 36 to 48 h. When transformations were performed under nonselective conditions, a spot transformation procedure was used^53^. For transformation, two piliated colonies were resuspended in 100 μl GCP containing 200 mM MgCl_2_, 0.42% NaHCO_2_, and Kellogg’s supplement. The cell suspension was diluted 1:10, and additional 3-fold serial dilutions were carried out 9 times. An aliquot (5 μl) of each suspension was spotted onto a GCK agar plate. Five microliters of DNA was added to each spot. After incubation overnight at 37 °C with 4% CO_2_, individual colonies were isolated and struck for isolation on GCK agar plates. The next day, individual colonies were inoculated onto GCK and the appropriate antibiotic -containing GCK agar plates. This procedure was repeated until antibiotic -sensitive colonies were obtained. The correct replacement of the desired DNA fragment by the transformation process was verified by PCR amplification of the desired region and restriction digestion analysis of the PCR amplicon or direct DNA sequencing of the PCR amplicon. Generally, each transformant was passaged twice for each strain construction/identification.

### Construction of *N. gonorrhoeae* FA1090 lacking all filamentous phages coding sequences

The general strategy used for cloning can be found in supplemental Figure S1. To delete the filamentous phage encoding DNA, primers were designed to amplify a sequence around the coding region. The flanking sequence was always about 1000 bp long and designed in such way that it always contained at least one DNA transformation uptake sequence^54^. The DNA fragments flanking the left and right end of each filamentous phage DNA sequence were obtained by PCR amplification and digested with restriction enzymes whose recognition sequences had been incorporated into the PCR primers. The same restriction enzymes were used to digest plasmid DNA encoding the particular antibiotic resistance cassette. To identify transformants carrying the NgoΦ6 deletion, DNA encoding the kanamycin cassette from pKRP11 was cleaved with HindIII and PstI, purified from the agarose gel and ligated with two phage-flanking sequences. The ligation mixture was used as a template to amplify the two ligation products and the resulting amplicon used to transform *N. gonorrhoeae* FA1090. Kanamycin resistant transformants were tested by a PCR method to verify the deletion of the DNA sequence encoding NgoΦ6. One of such transformant named FA1090ΔΦ6 was used for further construction. To allow for the selection of transformants carrying the NgoΦΔ7 deletion, a chloramphenicol cassette was obtained from plasmid pACYC184 digested with the appropriate enzymes and the resulting DNA fragment ligated with the two phage-flanking sequences. The ligation products were used to obtain the appropriate amplicon containing two phage-flanking sequences and then transformed into *N. gonorrhoeae* FA1090ΔΦ6 cells. Chloramphenicol resistant transformant colonies were tested by PCR to verify the deletion of DNA sequence encoding NgoΦ7. The chloramphenicol cassette was removed from this transformant by ligating the two flanking sequences, with subsequence transformation and identification using the spot transformation method. One of such transformant named FA1090ΔΦ6ΔΦ7 was used for further construction. In similar way the mutant lacking NgoΦ9 DNA sequence was isolated. The ligation products were transformed into *N. gonorrhoeae* FA1090ΔΦ6ΔΦ7 cells. Chloramphenicol resistant colonies were isolated and checked by PCR method for the lack of NgoΦ9 DNA sequences. One of such transformant named FA1090ΔΦ6ΔΦ7ΔΦ9 was used for further constructions. To delete NgoΦ8 DNA, the kanamycin cassette originally occupied by NgoΦ6 was deleted by a spot transformation procedure. The correct replacement of the desired DNA fragment by the transformation process was verified by PCR amplification of the desired region and restriction digestion analysis of the PCR amplicon followed by direct DNA sequencing of the PCR amplicon. In this way the strain with deleted NgoΦ6, Φ7 and Φ9 phages but sensitive to kanamycin was obtained. To delete NgoΦ8 the kanamycin resistant gene from pKan4 was amplified and ligated with the NgoΦ8 flanking DNA sequences. The ligation products were transformed into *N. gonorrhoeae* FA1090ΔΦ6ΔΦ7ΔΦ9 cells. Kanamycin resistant colonies were isolated and checked by PCR method for the lack of NgoΦ8 DNA sequences. One of such transformant named FA1090ΔΦ6789 was used in further experiments.

### Construction of recombinant plasmids and purification of ORF9 protein

To express ORF9, we constructed a derivative of plasmid pET28a containing the NgoΦ6 phage *orf9* gene. A fragment of *N. gonorrhoeae* (coordinates 1082262-1083347) was amplified using primers 0F16NheI and 9FQER and cloned into the NheI and HindIII sites. The PCRs were carried out using Pfu DNA polymerase (Fermentas) according to the manufacturer’s recommendations. To express and purify the ORF9 protein a single colony of *E. coli* BL21(DE3)(pET28a::*orf*9:HisTag) generated by fresh transformation was used to inoculate 100 ml of LB broth containing kanamycin. Cultures were incubated at 37 °C, and when the optical density of the culture at 600 nm reached 0.6, isopropyl-β-d-thiogalactopyranoside (IPTG) was added to a final concentration of 1 mM. Incubation was continued at 25 °C for an additional 2.5 h. The cells were collected by centrifugation, and the bacterial pellet was resuspended in 10 ml of buffer containing 50 mM NaHPO_4_, 300 mM NaCl, 20 mM imidazole, 10 mM β-mercaptoethanol, 0.1% Tween 20, 55 μM phenylmethylsulfonyl fluoride (PMSF), and 1 tablet of EDTA-free complete Mini (protease inhibitor cocktail) (Roche Diagnostics). After sonication, the cellular debris was removed by centrifugation at 40,000 ×  g for 1 h and the supernatant applied to a 3-ml Ni-nitrilotriacetic acid (NTA) agarose column previously equilibrated with 100 ml of the above-described buffer. The column was washed with 250 ml of buffer containing 50 mM NaHPO_4_, 300 mM NaCl, 35 mM imidazole, and 10% glycerol. The proteins were eluted with a gradient of imidazole (50 mM to 0.5 M) in the same buffer. ORF9 protein eluted at 0.2 to 0.25 M imidazole. The homogeneity of the purified protein was determined by electrophoresis on a 15% SDS-PAGE gel. The amount of purified protein was determined by using Bradford Reagent (Sigma-Aldrich), with bovine serum albumin (BSA) as the protein standard. The yields of purified protein were ~3 mg/liter. PageRuler prestained protein ladder (170, 130, 100, 70, 55, 40, 35, 25, 15, and 10 kDa) (Fermentas) was used as the protein molecular mass marker.

### Cell-fractionation

An *E. coli* BL21(DE3) strain carrying pET28a::*orf*9::HisTag was grown in LB media supplemented with kanamycin. Cultures were diluted 1:50 in 200 ml of fresh media and ORF9 production was induced as described above. Bacteria were harvested by centrifugation at 6000 rpm in a Beckman JA20 rotor at 4 °C for 20 min. The pellet was resuspended in 30 ml of PBS in the presence of β-mercaptoethanol (final concentration of 140 nM). Bacteria were harvested again and suspended in 10 ml of PBS buffer. After sonication (20 pulses for 15 sec, amplitude 30%), cell debris was collected by centrifugation (12,500 rpm, 10 min in a Beckman JA20 rotor at 4 °C) and the supernatant was centrifuged at 40 000 rpm (Beckamn Ti60 rotor at 4 °C for 30 min). The precipitate was suspended in 3 ml of PBS buffer containing 2% of N-laurylsarcozine. The supernatant containing outer membrane proteins were stored at −20 °C. The pellet containing inner membrane proteins was suspended in 1 ml of PBS buffer and stored at −20 °C. For the isolation of the periplasmic proteins, 2 ml of induced *E. coli* culture was centrifuged (at 6000 rpm in a Beckman JA20 rotor at 4 °C for 20 minutes) and the pellet resuspended in PBS and vortexed. Chloroform (20 μl) was added and the cells suspension vortexed again. The sample was incubated at room temperature (20 min) and 200 μl of Tris-HCl buffer (10 mM pH 7.0). After centrifugation (12,500 rpm in a Beckman JA20 rotor at 4 °C), the supernatant containing periplasmic proteins was stored at −20 °C.

### Phage and phagemid particles preparation

Overnight cultures were diluted 50-fold into 200 ml of an appropriate medium and grown with shaking at 30 °C or 37 °C until the optical density at 600 nm (OD600) was 1. In some experiments, mitomycin C (final concentration, 0.3 μg/ml) was added, and incubation continued with shaking overnight. If mitomycin C was not added, cells were simply grown overnight. Bacteria were collected by centrifugation (20 min at 7,000 rpm), and the supernatant was filtered through a 0.8-μm filter. The filtrate was mixed with 1/5 volume of a solution containing 20% polyethylene glycol (PEG-8000) and 2.5 M NaCl and kept at 4 °C overnight to precipitate the phage particles. The precipitate was collected by centrifugation, dissolved in 4 ml of phosphate-buffered saline (PBS), and treated with DNase I and RNase A (25 μg/ml each) for 3 h at 20 °C. Two types of phage particles were further prepared. Type A particles were obtained by purification of phage suspension on a QMA-Sephacell column (2 cm by 10 cm). Type B particles were obtained by sequential centrifugation of the PBS phage suspension at 4000 rpm for 10 min and 19 000 rpm for 120 min at 4 ^o^C in an SS34 rotor. The precipitate was dissolved in 1 ml of 50 mM Tris-HCl buffer, pH 7.5. This step was repeated twice.

### Production of polyclonal antisera

An overnight culture (1ml) of STΦ6 was used to inoculate 100 ml of LB broth containing DAP and ampicillin and incubated at 37 °C with shaking overnight. After this time, cells were collected by centrifugation (Beckman rotor, 6000 rpm, 19 min at 4 ^o^C) and the pellet suspended in 20 ml of cold BSG buffer (PBS buffer containing 20% glycerol). The cells were centrifuged and the pellet suspended in 10 ml of BSG buffer and diluted in such a way that the final concentration of cells was about 10^8^/ml. The cells suspension was stored at −70 °C until needed. This cell suspension was used in EUROGENETIC S.A. Liege Belgium, for immunization of three rabbits according to their standard protocol (ref. no AS-PNOR-3MORAB). In this protocol vaccine was introduced four times *per os* at days 0, 14, 28 and 56 day using 0.5 ml of bacterial cell suspension per animal per immunization. Sera was collected at day 0, (before immunization), at day 38, at day 66 and at 87 day. All animal work performed at the CER Groupe was carried out in accordance with the 2010/63/EU directive on the protection of animals used for scientific purposes. The protocols were approved under reference CE/SANTE/E/001 by the CER ethical licensing committee.

### Determination of antibodies against ORF9 protein by dot spot ELISA

ORF9 (4 μl) of protein at concentration 5 μg/ml in carbonate buffer (50 mM sodium bicarbonate, 0.03 M sodium azide, pH 9.6) was spotted in triplicate onto a nitrocellulose membrane and dried at room temperature. The membranes were incubated with various dilutions of sera. To detect antibody binding, Goat Anti-Rabbit IgG (H + L), Alexa Fluor® 488 conjugate Antibody was obtained from Sigma-Aldrich (St. Louis, MO). For all immunological binding experiments, our positive control was mouse reference antibodies (Sigma-Aldrich Cat no. A493, 5 μg/ml) and the negative control contained only buffer used for immobilization. After three washes with 20 ml of PBS buffer, the membranes were blocked with 4% (w/v) nonfat milk in PBS at 25 °C for 1 h. Following the washes, different dilution of sera collected after 0, 38, 66 and 87 days, in PBS with 4% (w/v) of nonfat milk were incubated at 25 °C for 1 h. The membranes were washed three times with PBS and incubated with secondary antibodies at room temperature for 1 h. Secondary antibodies included alkaline-phosphatase -conjugated goat anti-rabbit IgG (ref. no M1421, Sigma-Aldrich, USA) diluted in PBS at 1:5,000. Secondary antibody was removed, and the membranes washed four times for 5 min with PBS + 0.1% Tween-20 and once with PBS. Membrane were soaked in 20 ml of detection buffer (AP, 0,1 M Tris-HCl pH 9,5; 0,1 M NaCl; 5 mM MgCl_2,_ pH 9,5) containing 20 μl of NBT BCIP (Sigma, USA) for 30 min at room temperature in darkness. The reaction was stopped by intensive washing with distilled water and the left for drying. The amount of protein contained in each spot visualized on the membrane and quantified using GeneTools GBox (Syngen) program. The intensity of the each spot was expressed as the increase of the intensity compare to the negative control where spotting of protein was omitted.

### Determination of the level of antibodies against *N. gonorrhoeae* cells by the Dot blot ELISA

Determination the level of anti-*N gonorrhoeae* specific IgG and IgA antibodies in the rabbit sera was based on the method by Afonina *et al.*^55^ and Cole and Jerse^56^. *N. gonorrhoeae* cells (2 μl of 10^8^ cells/ml diluted in PBS) were spotted in triplicate onto 10 × 50 mm strips of nitrocellulose and allowed to air dry. The membrane strips were blocked by incubating in PBS containing 1% nonfat milk as the blocking solution for 3 hr at room temperature. Sera were diluted in blocking buffer and incubated with shaking for 1 hour at room temperature. Membranes were washed four times for five minutes with PBS +0.1% Tween-20. Membranes were incubated 1 hr at room temperature in mouse monoclonal Anti-Rabbit IgG (gamma-chain specific)-Alkaline Phosphaase Goat-anti-rabbit secondary antibody) diluted 1:5000 in blocking solution (Sigma-Aldrich, USA ref. no 056K4798) or goat polyclonal Anti-Rabbit IgA (gamma-chain specific)-Alkaline Phosphaase) secondary antibody diluted 1:5000 in blocking solution (Abcam ref. no ab97187) and incubated for 1 h at room temperature. Secondary antibody was removed, and the membranes washed four times for 5 min with PBS +0.1% Tween-20 and once with PBS. Membrane were then soaked in 20 ml of detection buffer (AP, 0,1 M Tris-HCl pH 9,5; 0,1 M NaCl; 5 mM MgCl_2_ pH 9,5) containing 20 μl of NBT BCIP (Sigma, USA) for 30 min at room temperature in darkness. The reaction was stopped by intensive washing with distilled water and the left for drying.

The amount of protein contained in each spot was detected and quantified as described above. The quantity of specific antibodies bound to the spots was determined by comparing the optical density of specific anti-*N. gonorrhoeae* IgG or IgA antibodies bound to the spots to a standard curve obtained with known quantities of purified mouse IgG or IgA reference antibodies.

### Indirect fluorescent antibody staining

Indirect fluorescent antibody staining was carried out essentially described by Lin *et al.*^57^. In brief, *N. gonorrhoeae* FA1090 cells from freshly grown plates were suspended in TBS to a density of 2 × 10^8^/ml. Cells were centrifuged and the pellet resuspended in 1 ml of TBS. Cells were centrifuged again and suspended in 1 ml of TBS containing 4% of nonfat milk. The sera obtained after 66 days from immunization of rabbits or before immunization were added in concentration of 1:500 and the cells were incubated for 90 min at RT. Cells were washed three times with TBS, suspended in 1 ml of TBS and secondary antibody Alexa Fluor Cy3 R goat anti-rabbit IgG (H + L) (Invitrogen, Carlsbad, California) (1: 250) were added. After 30 min incubation at room temperature cells were washed two times with TBS and analyzed with a Nikon Upright Fluorescence Microscope Eclipse E800 equipped (60 × NA 1.40 Plan Apo) and for Nomarski contrast CDC Orca-ER Hamamatsu camera. A system for Image Processing and Analysis Lucia General 4.82 and 5.0 – software for image capture by CCD cameras and simple measurements were used for analyzing data.

### Flow cytometric analysis

Flow cytometry was performed essentially as described by Price *et al.*^10^. All buffers were filtered through a 0.22-μm filter (Millipore) to remove particles that could interfere with the analysis. Non-piliated FA1090 and FA1090ΔΦ6789 were incubated overnight on GCB agar plates containing Kellogg’s supplement, at 37 °C in a 5% CO_2_ atmosphere. Single colonies were passaged onto GCB plates. Bacteria were harvested into PBS + 0.05% Saponin (Sigma) to a density of ~2 × 10^8^ CFU/ml. One ml aliquots of the cell suspension were collected by centrifugation at 10,000 × g for 2 min and the pellets washed twice with PBS + 0.05% Saponin. Bacteria were fixed with 1% paraformaldehyde in PBS for 30 min at room temperature while protected from light, washed twice with PBS, resuspended in PBS + 4% of nonfat milk (Sigma) and incubated for one hour at RT. After two washes with the same buffer, cells were suspended in PBS + 4% of nonfat milk containing appropriate rabbit sera and incubated for one hour at RT. Following one wash with the same buffer, bacteria were incubated with an AlexaFluor Cy3 goat anti-rabbit IgG (Invitrogen, Carlsbad, California) secondary antibody (Molecular Probes) for 30 min at RT. After one wash with the same buffer, cells were resuspended in one ml of buffer and filtered through a 35 μm nylon mesh to remove any flocculent debris. Antigen-antibody binding was measured by flow cytometry as median fluorescence intensity with a Coulter EPICS XL-MCL flow cytometer.

### Serum bacterial assay

The serum bacterial assay was essentially carried out as described by Li *et al.*^7^. Gonococcal strains FA1090, F62 and MS11 were resuscitated on GC chocolate agar directly from a freezer stock. The plates were incubated at 37 °C and 5% CO_2_ for 16 to 18 hrs and the bacteria resuspended in prewarmed GCK medium (37 °C). The cell suspension was adjusted to about 3 × 10^4^ CFU/ml. Rabbit sera were pooled (n = 4) and heat inactivated at 56 °C for 30 min. A total of 50 μl of serially diluted serum samples in PBS and 40 μl of bacterial suspension were mixed and incubated at 37 °C and 5% CO_2_ for 15 min. Undiluted normal human serum (4 μl) was added into the mixtures to supply the complement sources, and the incubation was continued for an additional 45 min. Samples were plated onto three plates of GCK agar and incubated for approximately 24 h, and colonies enumerated. Titers were calculated as the reciprocal of the dilution that resulted in >50% killing as compared to CFU detected in the presence of antibody but in the absence of human sera. Assays were performed at least in triplicate.

## Additional Information

**How to cite this article**: Piekarowicz, A. *et al.* Oral Immunization of Rabbits with *S. enterica Typhimurium* Expressing *Neisseria gonorrhoeae* Filamentous Phage Φ6 Induces Bactericidal Antibodies Against *N. gonorrhoeae. Sci. Rep.* **6**, 22549; doi: 10.1038/srep22549 (2016).

## Supplementary Material

Supplementary Information

## Figures and Tables

**Table 1:** Properties of NgoΦ6^a^.

ORF	Gene	Coding sequence	Amino acids	Predicted Mass
1	NGO1146	1230	409	46648
2	NGO1145	312	103	10979
3	NGO1144	204	67	7765
4	NGO1143	219	72	7539
5	NGO1142	279	92	10435
6	NGO1141	318	105	12428
7	NGO1140	1524	507	56037
8	NGO1139	297	98	10619
9	NGO1138	1086	361	40801
10	NGO5940	360	119	14254
11	NGO1137	963	320	36337

^a^Properties of NgoΦ6 were predicted from the DNA sequence of *N. gonorrhoeae* strain FA1090 with the GenBank accession number AE004969.1.
