# Supplementary material for: Oral Immunization of Rabbits with S. enterica Typhimurium Expressing Neisseria gonorrhoeae Filamentous Phage Φ6 Induces Bactericidal Antibodies Against N. gonorrhoeae
Source: Sci Rep. 2016 Mar 4;6:22549. doi: 10.1038/srep22549 (PMC4778046; doi:10.1038/srep22549)

Oral Immunization of Rabbits with *S. enterica Typhimurium* Expressing *Neisseria gonorrhoeae* Filamentous Phage Φ6 Induces Bactericidal Antibodies Against *N. gonorrhoeae*

Andrzej Piekarowicz[1]*, Aneta Kłyż[1], Michał Majchrzak[1], and Daniel C. Stein[2*]

[1]Department of Virology, Institute of Microbiology, Faculty of Biology, University of Warsaw, Warsaw,

[2] University of Maryland

Department of Cell Biology and Molecular Genetics

College Park, MD 20742

Supplemental TABLE 1

Primers used in this study

Primer	Sequence (5′ → 3′)	Use
ORF9F	AACTTTCGACGTGTCCGGGAACTTATGGAGACGGA	Cloning of ORF9 of NgoΦ6
ORF9R	AAGGCGCGGTGCTGATTGTTGGCGAAGCGCACTA	into pET28a
PhageΦ9R	TTATTGATTCAATCGGTGTCTTTCC	Detection of the presence
PhageΦ9F	TTACGCCGATTTGTAACGCGATGGA	of phage NgoΦ9
PhageΦ8R	ATGGGCTTAAAGTGTCTGCCCGAAA	Detection of the presence
PhageΦ8F	TTACGCCGATTTGTAACGCGATGGA	of phage NgoΦ8
PhageΦ7R	ATGATGACGATTTCATACGCGCGGC	Detection of the presence
PhageΦ7F	TTACGCCGATTTGTAACGCGATGGA	of phage NgoΦ7
PhageΦ6R	TTTCATTTCCGGATTCCCACTTTCG	Detection of the presence
PhageΦ6F	ATGCGTAACGCCGTAGGATTGGATA	of phage NgoΦ6
Φ6LF	TCCGAACCTTTGAAGCAA	Amplification of the
Φ6LR	CACTAAGCTTGGGATTACCCTTTATATGCGGATAT	left flank of NgoΦ6
Φ6RF	CACTCTGCAGCAGCGTTGCGAGGCTTAAGC	Amplification of the
Φ6RR	GCCGGCCGCCCCGGACT	right flank of NgoΦ6
Φ7LF	GTCCTCGCCTCGCTCG	Amplification of the left
Φ7LR	GTGAAGCTTTTGCCGGTGTATTGGTAGG	flank of NgoΦ7
Φ7RF	CACTCTGCAGAAATCGGTAAATTTGCGT	Amplification of the right
Φ7RR	AGGGTTGGGCGGGACCCATTCCCCTTCTC	flank of NgoΦ7
Φ8LF	GACCCATTCCCCTTCCTC	Amplification of the left
Φ8LR	GTGAAGCTTTCCCGAAAGGAAAAAAGG	flank of NgoΦ8
Φ8RF	CTCACTGCAGTATATCGATCCATCC	Amplification of the right
Φ8RR	GATGATGACGATTTCATAC	flank of NgoΦ8
Φ9LF	TATTAAACACATAAAAACAAAGGA	Amplification of the left
Φ9LR	GTGAAGCTTCGATAAATCCCGAAAGG	flank of NgoΦ9
Φ9RF	CTCACTGCAATAAAATTCAACAAAATT	Amplification of the right
Φ9RR	GTTCGGCGGTGTTCGCCCG	flank of NgoΦ9
Omega-PstFR	CAGTCTGCAGGAGTTAAGCCGCGCCGCGAA	Amplification of the
Omega-HindIIIF	GACCTCGAGTTGCAAACCGAAGCTTGTG	spectinomycin cassette
0F16NheI	CTAGCTAGCATTTATCTGTTTACGGGAACAT	Cloning of Orf9 into
9FQER	GCAAGCTTTTAGGACTTTCCGCCCATTC	pET21A
ChlorF	ATCGTTTAAACGTTGATCGGCACGTAAGAG	Amplification of the
ChlorR	ATCGTTTAAATAGCACCAGGCGTTTAAGGG	chloramphenicol cassette
KanF	CACTAAGCTTTATCTGGACAAGGGAAAACGC	Amplification of the
KanR	CACTCTGCAGGGTGGAATCGAAATCTCGTGA	Kanamycin cassette

Fig.S1. Schematic of method used to delete NgoΦ6, NgoΦ7, NgoΦ8 and NgoΦ9 from FA1090. (A) Locations of the filamentous phage sequences on the FA1090 genome as determined by (28). (B) General scheme for removing phage sequences. Two amplicons (L-left and R-right) flanking each phage sequences were obtained by PCR and after digestion with HindIII (left flank DNA) and PstI (right flank DNA) ligated with the donor of antibiotic resistance cassette to produce a fragment (C). The ligation product containing antibiotic cassette integrated into left and right flanks of phages DNA was amplified by PCR using appropriate primers. Panel D indicates that the integration of the amplified DNA after *N. gonorrhoeae* transformation and selection of antibiotic resistant transformant (Panel E).

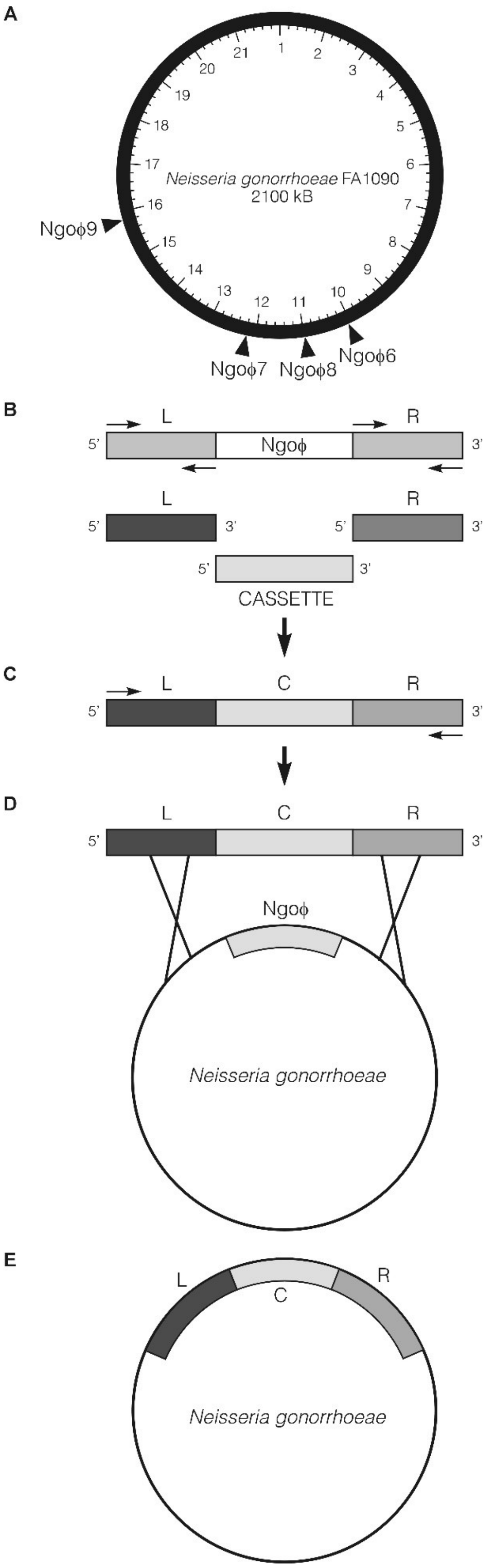
A
Neisseria gonorrhoeae FA1090
2100 kB
1
2
3
4
5
6
7
8
9
10
11
12
13
14
15
16
17
18
19
20
21
Ngoφ9
Ngoφ7
Ngoφ8
Ngoφ6
B
L
R
5'
Ngoφ
3'
L
R
5'
3'
5'
3'
5'
3'
CASSETTE
C
L
C
R
5'
3'
D
L
C
R
5'
3'
Ngoφ
Neisseria gonorrhoeae
E
L
C
R
Neisseria gonorrhoeae

Construction of Figure 1:

Lanes M and 1

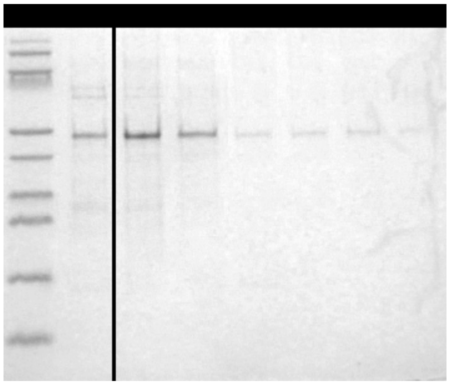

Lane 2

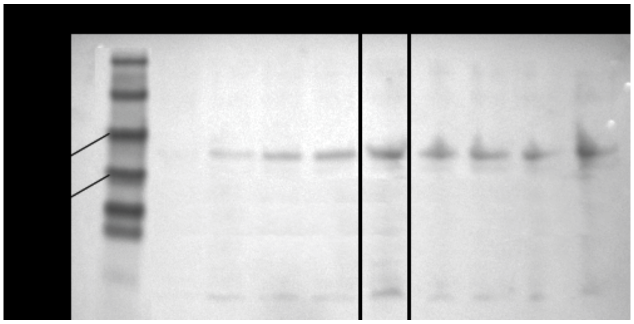

Lane 3, 4 and 5

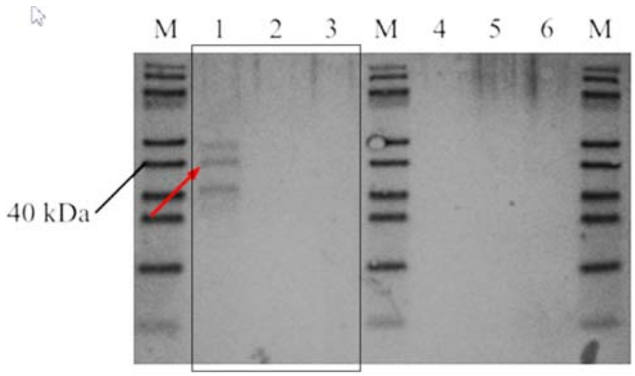

Final image:

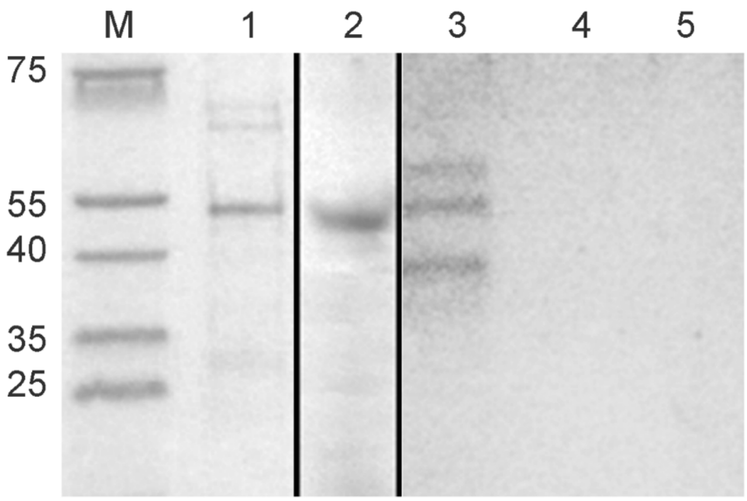

Supplement: Supplementary Information [file srep22549-s1.pdf]
